# Implementation of disaster risk reduction and management policies in a school setting in Lao PDR: a case study

**DOI:** 10.1186/s41182-018-0124-7

**Published:** 2018-12-12

**Authors:** Kethsana Kanyasan, Daisuke Nonaka, Athithouthay Chatouphonexay, Paul Michael Hernandez, Sengchanh Kounnavong, Jun Kobayashi

**Affiliations:** 1 Department of Global Health, Graduate School of Health Sciences, University of the Ryukyus, Uehara 207, Nishihara-cho, Okinawa 903-0215 Japan; 2 Faculty of Education, National University of Laos, North 13 Road, Dongdok Campus, 7322 Vientiane Capital, Lao People’s Democratic Republic; 3 Department of Environmental and Occupational Health, College of Public Health, University of the Philippines Manila, 625 Pedro Gil Street, Ermita, 1000 Manila, Philippines; 4 Lao Tropical and Public Health Institute, Ministry of Health, Ban Kaongot, Samsenthai Road, Sisattanak District, Vientiane Capital, Lao People’s Democratic Republic; 5 Japanese Consortium for Global School Health Research, Uehara 207, Nishihara-cho, Okinawa 903-0215 Japan

**Keywords:** Disaster risk reduction, Policy implementation, Fire disaster, School health, Laos

## Abstract

**Background:**

Lao People’s Democratic Republic (Lao PDR) formulated the National Strategic Plan for Disaster Risk Management to reduce risks to the communities. This plan was eventually integrated into the school curriculum, but its implementation has never been evaluated. This study aimed to clarify the present situation to inform better implementation strategies on disaster risk reduction and management in a school setting focused on fire disasters in Lao PDR.

**Methods:**

A case study was conducted in Vientiane and five provinces in 2017. Key informant interviews were conducted among 52 policy implementers from the Disaster Management Committee (DMC), the education, and fire service sectors at national, provincial, district and school levels. Observations were done among eight secondary schools, and questionnaires were answered by 869 grade 7 students. Interview transcripts underwent content analysis using the 12 influential components of successful policy implementation and the 3 pillars of comprehensive school safety framework. The level of student knowledge on fire prevention and response was examined.

**Results:**

Three themes emerged: policy content and dissemination, factors which affect policy implementation, and impacts of policy implementation facilitating factors include effective coordination and ownership among the national DMC members for scaling up disaster risk reduction (DRR) activities, and strong support from the central government. Barriers include unclear provisions in the national legislation, unclear mandates especially on leading the program, poor monitoring system, insufficient human resources, and lack of public-private partnerships. All the study schools conducted DRR classes and designated a disaster assembly point. More than 80% of the students correctly answered items on fire response.

**Conclusion:**

The policy was widely disseminated and implemented in all levels across sectors among the study sites except for some rural areas. Although there is a lack of national legislation and clear mandates, strong leadership, and ownership of the implementers facilitated policy implementation. All the study schools conducted fire prevention activities. Most students knew how to appropriately respond to fire. A comprehensive school-based DRR program would be beneficial in improving student knowledge and practices on DRR.

## Background

**Fig. 1:**
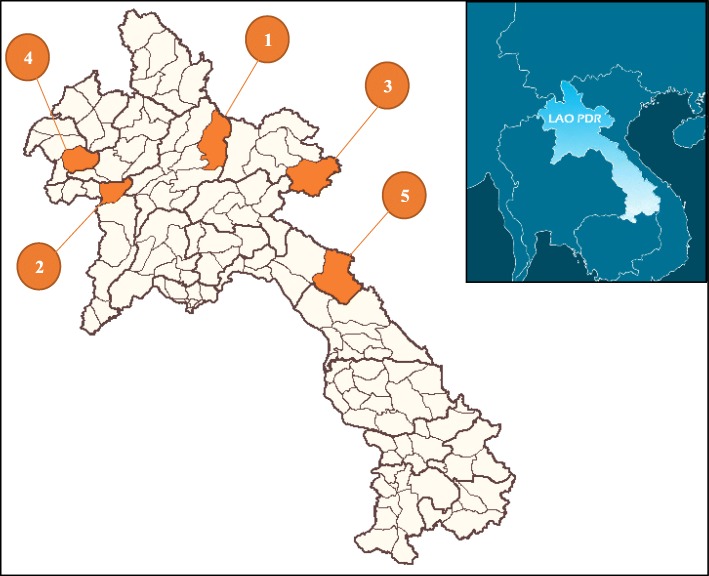
The location of the Lao PDR and study areas. The study included (1) Viengkam District, Laung Prabang Province, (2) Hongsa District, Xayaboury Province, (3) Xamtai District, Houaphanh Province, (4) Pha Udom District, Bokeo Province, (5) Khamkuet District, Bolikhamxay Province

Recently, Asian tropical countries including Lao People’s Democratic Republic (Lao PDR) have faced a large number of natural and man-made disasters, which have massive negative effects on the people in the affected countries [1]. Lao PDR is a landlocked, lower-middle income country surrounded by Vietnam, China, Myanmar, Thailand, and Cambodia (Fig. 1). In 2015, its population was 6.5 million with 80% in the rural areas. It is estimated that 85% to 90% depend on subsistence farming [2].

The Lao PDR National Assessment Report on Disaster Risk Reduction showed that disasters killed 380 people with estimated economic damages at US$ 849 million from 1990 to 2012 [3]. Also, disasters affected 320 schools across the country from 2013 to 2016, resulting to 6 deaths and 52 injuries among students [4]. Among the disasters, fire most frequently occurs and has the greatest impact in terms of lives lost, injuries, and economic losses in the country. From 2001 to 2016, there were 2134 fire incidents resulting to 61 deaths and 67 injuries [3].

**Fig. 2:**
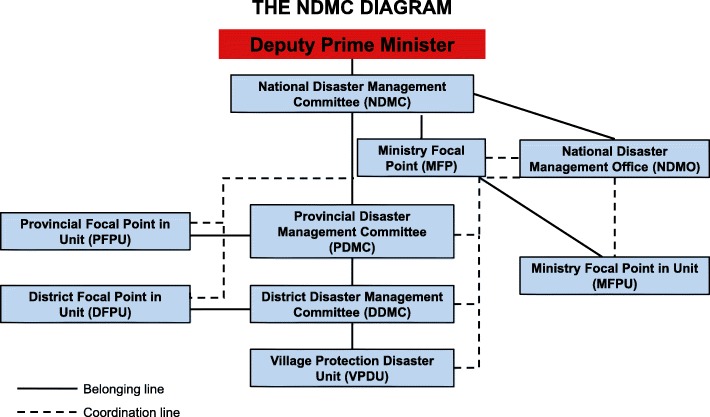
National Disaster Management Committee Diagram of Lao PDR

In August 23, 1999, a simple national government structure was built to guide the country’s disaster risk reduction and emergency response management. The government also created the National Disaster Management Committee (NDMC) which includes 13 Chiefs or Directors from various ministries. The committee is chaired by the Deputy Prime Minister (Fig. 2). The NDMC is tasked with coordinating early warning, preparedness, emergency response, and recovery activities. The National Disaster Management Office was assigned as the Secretariat to the NDMC, and a focal point structure was later established which are made up of NDMC members and units. Since then, committees and offices with formal lines of reporting have been established through the Provincial Disaster Management Committees, District Disaster Management Committees, and Village Disaster Management Committees [5]. According to a subsequent decree, the 097/MLSW, the National Strategic Plan for Disaster Risk Management 2003–2020 was developed and issued in 2003. The National Disaster Management Plan 2012–2015 was then issued in 2011. These plans provided the current policy framework for disaster management in the nation which aims to (1) reduce disaster risk to the communities and (2) to strengthen capacities of disaster management bodies at the national, local, community, and school levels [6].

As a member of the NDMC, the Ministry of Education and Sports (MOES) formed a project technical working group composed of senior officials from the National Research Institute for Educational Science, MOES, United Nations Development Program (UNDP) and Save the Children International (SCI). In 2007, the Disaster Risk Reduction (DRR) module was integrated into the existing natural and social science subjects and piloted in target schools. Similarly, textbooks for grades 3, 4, and 5 were developed to teach DRR [7]. The DRR curriculum, teacher’s guidelines, and the student textbooks were revised in 2014 by the National Research Institute for Educational Science in cooperation with SCI. SCI identified project schools to implement the program. The program included training of DRR teachers in grades 3 to 6 on how to use the revised materials and conduct DRR activities. A few schools also practiced drills and mock evacuations. For example, two schools in Khamkuet District (number 5 in Fig. 1) conducted DRR simulation exercises. In the communities where MOES and SCI work, there were also evacuations and drills. The village education committees were also established to ensure that DRR information is disseminated throughout the community [8]. The National School Health Policy was established in 2005 through a cooperation between the MOES and the Ministry of Health which supported efforts in DRR specifically on reducing risks at the school and improving the DRR capacity of teachers [9].

Policies and programs require active management to be successful. This involves measurement, analysis, consideration of feedback and complaints, evaluation and review, calibration, and adjustment [10]. Studies conducted in Thailand [11] and Vietnam [12] highlighted the importance of understanding the status of health policy implementation. Other studies show that schools have an important role in preparing children to face disasters in Thailand [13, 14] and Australia [15, 16]. Recent experiences in disasters have increased awareness at the highest levels of government and the National Assembly of Lao PDR as shown by one study: the study identified key strategies in improving disaster recovery efforts resource generation, institutional knowledge development, capacity-building, and institutional mandate and coordination [17]. No studies have assessed the implementation of DRR policies on prevention, preparedness, and response in Lao PDR.

Therefore, this study aimed to clarify the present situation to inform better implementation strategies on disaster risk reduction and management in a school setting focused on fire disasters in Lao PDR. It specifically aimed to (1) determine facilitating factors and barriers in disaster risk reduction and management at the national, provincial, district, and school levels; (2) determine DRR activities of selected schools; and (3) assess knowledge of grade 7 students on fire prevention and responses among project and non-project schools.

## Methods

### Study site and populations

The present study applied a case study approach [18, 19] and was conducted in Vientiane capital, Luang Prabang, Xayaboury, Houaphanh, Bokeo, and Bolikhamxay provinces in 2017. These provinces were selected because these had a higher number of fire disaster incidents at schools [3]. For each study province, one district was selected by the Provincial DMC. The study districts were (1) Viengkham District of Luang Prabang Province, (2) Hongsa District of Xayaboury Province, (3) Xamtai District of Houaphanh Province, (4) Pha Udom District of Bokeo Province, and (5) Khamkuet District of Bolikhamxay Province (Fig. 1). For each district, at least one secondary school that has experienced fire disaster was selected by the District DMC, making a total of eight schools (Table 1).

**Table 1:** Student number, fire experience, and location of study schools

School ID	Number of students	Fire experience	District and province
1	181	Yes	Viengkham District, Luan Prabang Province
2	130	No	
3	45	Yes	Hongsa District, Xayaboury Province
4	117	Yes	Xamtai District, Houaphanh Province
5	71	No	
6	129	Yes	Pha Udom District, Bokeo Province
7	130	No	
8	66	Yes	Khamkuet District, Bolikhamxay Province
Total	869		

#### Participants of the key informant interviews

Those who have roles and responsibilities on DRR for fires among schools as stated in the National Disaster Management Strategy were included [20]*.* There were 52 key informant interviews with DRR implementers who represented the three main groups of respondents: the Disaster Management Committee (DMC), the education, and fire service sectors. Four came from the national level (two from the education sector, one each from the DMC, and fire service sector). Also included were representatives of the same group of respondents from five districts and their respective provinces. From each of the eight selected schools, the school principal and a DRR teacher were also interviewed. Also, a representative from the two donor agencies was included (UNDP-Lao PDR and Save the Children International-Lao PDR).

#### Participants of school-based survey

The eight schools were grouped into two; those which partnered with Save the Children International (project schools) and those which did not (non-project schools). There were six project schools and two non-project schools. Grade 7 students from these schools were included. They are assumed to have undergone the DRR modules when they were in grades 3 to 6. There were 668 students from project schools and 201 students from non-project schools.

### Data collection

The investigators conducted key informant interviews, document reviews, archive record reviews, and school-based survey including both school observation and student knowledge assessment. Data collection was done between January to March 2017 and September to November 2017.

#### Key informant interviews

The study used a modified interview guideline based on the “Policy-Implementation Assessment Tool for Implementers and Other Stakeholders” developed by the United States Agency for International Development (USAID). The original interview guideline consisted of seven dimensions which were designed to capture the overall process of the policy implementation. This guide has been used for policy analysis in several lower-middle income countries [21]. The guide was translated to Lao and pre-tested among 47 grade 7 students at Demonstration Secondary School which is under the Faculty of Education, National University of Laos. The final tool included questions on policy content, plan, present activities, main outcome, facilitating factors and barriers, and the challenges in implementing DRR activities.

#### Document reviews

Documents related to DRR and management in Lao PDR written in either English or Lao were reviewed. The investigators requested the DRR implementers and donor agencies to provide the relevant documents related to DRR. Online documents were searched through Google using a combination of the following search terms: disaster, disaster risk reduction, policy implementation, fire disaster, school safety, school health, and health policy. The search was restricted to documents published between 2003 and 2017, as the national strategic plan for disaster risk management was developed in 2003. The documents contained any or a combination of the following information: policy development, policy implementation, and the actors involved in the implementation. Three national policies related to DRR, 1 fire law, 2 guidelines, 2 DRR curricula, 2 national DRR assessment reports, 2 project reports, 11 DRR activity plans, 7 DRR training records at each level and sector were identified. Data were triangulated across data sources through personal dialog with national task force members and external experts at the national level both from education and disaster risk reduction and management sectors.

#### Review of archive records

Archive evidence such as video recordings, photos, newspaper and magazine articles, and web pages that are related to DRR policy implementation in Lao PDR, especially on fire disaster, was collected. These included both published and unpublished materials in any format.

#### Assessment of student knowledge

A questionnaire was developed based on previous studies on fire emergency preparedness at schools in Tanzania and Kenya [22, 23]. The questionnaire includes five items on fire prevention and seven on fire response. The questionnaire was translated into Lao and pre-tested among students in one school. The questions were modified accordingly. The investigators oriented the participants about the survey. Students and their guardians were requested to give their assent and consent, respectively. Those who agreed to participate were asked to accomplish the questionnaire.

#### School observation

Observation was done using a checklist developed from the same studies [22, 23]. It aimed to determine the status of disaster management at the school level, such as presence of emergency assembly point and its accessibility, disaster risk reduction classes, fire simulation exercises, fire protection gears, circuit breakers, water hydrant within the school, water resources for fire response, and distance of the school building to fire hazards such as garbage pit, cafeteria, or restaurant.

### Data analysis

Content analysis was done on the data collected during the key informant interviews. Codes and categories were derived directly from the text data [24]. Results were triangulated using the document reviews and archive records. The framework of 12 key components proposed by Withman was used to identify influential components of successful policy implementation [25]. These include vision and concept, dedicated time and human resources, stakeholder ownership and participation, team training and coaching, cross-sector collaboration, champions and leaders, data-driven decision-making, administrative and management support, adapting to local concerns, attention to external forces, critical mass and supportive norm, and stage of readiness [25].

The Comprehensive School Safety Framework was also used to determine if DRR activities have been implemented at the school. Its pillars are safe learning facilities, school disaster management and risk reduction and resilience education [26]. A large number of studies pertaining to disaster education have used this framework for policy analysis, including research on policies for disaster risk reduction [27–29].

Coding rules, codes, subcategories, and categories were then developed. The steps were repeated until sufficient coding consistency was achieved [30]. Finally, categories were classified into themes.

Results of the school observation were summarized in a matrix. Frequencies and percentages for the results of the questionnaire-based survey were computed. For the survey, Fisher’s exact test was used to determine if there is a significant difference in the knowledge between the two school groups. A *p* value < 0.05 was considered statistically significant. Statistical analyses were performed using SPSS version 24.

## Results

The DRR and management policy was disseminated and implemented at all levels across the sectors in most areas. The facilitating factors and barriers were summarized into three themes, namely, (1) policy content and dissemination, (2) factors which affect policy implementation, and (3) impacts of policy implementation (Table 2).

**Table 2:** Themes, categories, and sub-categories generated

Themes (3)	Categories (12)	Sub-categories (6)
Policy content and dissemination	Policy content, policy dissemination	
Factors which affect policy implementation	Legislation Leading agencies Decentralization and coordination Leadership Resources Program training Stakeholders Program evaluation Champion	Human Financial Infrastructure and equipment supply Monitoring Reporting Feedbacks
Impacts of policy implementation	Impact on school and community	

### Theme 1. Policy content and dissemination

The policy addressed most of the relevant issues, but it poorly covered the issue of man-made disasters. Among the issues covered, some were not applicable to Lao PDR. A representative of the education sector said, “The DRR curriculum was adopted from the general DRR curriculum designed for the Southeast Asia region. Hence, the content included volcanic eruption and tsunami. In reality, there are no volcanoes and seas in Lao PDR.”

The policy was disseminated through dissemination meetings, trainings, workshops, and mass media including a television program, radio and newspapers, seasonal notifications through village sound systems and leaflets, school activities, and community visits. These dissemination activities were done in urban communities but only in a few rural communities. Also, there is no specific budget for dissemination activities across all areas.

### Theme 2. Factors which affect policy implementation

Most of the respondents opined that there was no national legislation for DRR. Currently, the national legislation is being drafted by the National Disaster Management Office. However, the NDMC members in each sector were tasked to establish their own legislation for DRR. For example, MOES developed policies for DRR in the education sector and was established last February 2017.

The leading agency was frequently changed in the past two decades as this position was not fixed in the policy. A representative of the donor agencies said, “Sometimes, we get confused coordinating because the leading agency in the government frequently changes.”

Most of the participants acknowledged that the decentralization of decision-making and coordination system within DMCs had improved. One representative from a provincial DMC said, “We can work more easily and faster compared to before because we have the disaster management unit from the provincial up to the village level. Also, the lower disaster management units were empowered as they can make decisions based on the guidelines.” In contrast, there is a need to improve mid-level coordination among sectors. One representative from a district DMC mentioned, “There were so many members of district DMC. But in reality, only the Ministry of Labor and Social Welfare and Ministry of Natural Resources and Environment did the duties.”

Almost all respondents in each sector reported that their leaders gave them adequate support. A representative of one provincial DMC mentioned, “Our leader worked closely with us. He gave us suggestions when we faced problems, advocated us to attend the trainings on DRR management, and issued the official documents that facilitated our work.”

Human, financial, infrastructure, and equipment supply were perceived insufficient in each sector across levels. The representative of a provincial DMC said, “The responsible persons lack knowledge and skills on DRR management even though they have attended trainings. Also, there was no specific budget for DRR activities and unclear mechanism of accessing funds across levels. Moreover, collecting disaster information at the local level is hard because computers are lacking.” There was also a lack of DRR teachers. Some of the teachers transferred to other workplaces which affected implementation at schools.

The Xayaboury Province overcame financial limitations by establishing a disaster emergency response fund that was approved by the provincial governor. This fund was based on the set of standards of contributions from the government, non-governmental organizations, businesses, and the community with a target budget of 60,000 USD/year. At the school level, there was a financial support from the parents not only for DRR but for other activities. As one school principal said, “We also received financial support from the students’ parents as agreed during the community and school meetings. For this year, we got 50,000 Kip (8.5 USD) per student.”

Almost all respondents said that they received training and capacity building on specific topics in DRR which they acknowledged as useful. However, they recommended that the training be continuous and frequent, and include more participants. The conduct of fire service trainings in Hongsa District served as a model in collaborating with the private sector. The Hongsa Thermal Power Company*,* which is a local private company in the district, organized trainings twice a year. The Hongsa Thermal Power Company invites the Hongsa Fire Service to attend the trainings. A Hongsa firefighter commented that the training was good and well-organized. He said, “After the training, we were able to use fire equipment more effectively.”

All respondents said that monitoring, reporting, and feedback are important to improve policy implementation. At the national level, evaluation was done using the Hyogo framework criteria. In contrast, evaluation has not been conducted at provincial and district levels. As one provincial DMC member said, “We have never conducted an evaluation. We have to report our DRR activities to the central government, but the report has no criteria or indicator, and feedback from institutional leaders was not shared across organizations.”

Regarding stakeholder involvement, there was a good engagement among government and donor agencies such as UNDP-Lao PDR, SCI, and Red Cross–Lao PDR. An NDMC representative said, “Thirteen sectors of the central government were involved in policy implementation. Moreover, some NGOs and INGOs were involved.” However, there were limited public-private partnerships in some study areas.

Among the five study provinces, Xayaboury Province was successful in implementing the DRR policy through strong leadership, ownership, and coordination. The representative of the provincial DMC said, *“Our leader strongly supports us by establishing the regulations with clear and*, delineated tasks for each sector. These regulations were approved by the provincial governors. We also have an emergency fund from the contributions of government staff, private sector, business owners and community. Each sector was assigned DRR areas to promote their responsibility and ownership in the district level.” Also, a fire simulation exercise was conducted at the school and the community. The fire equipment was set up at the government office, hospital, factory, company, guesthouse, and some houses in the province. Moreover, the trainings by the fire service were supported by Hongsa Thermal Power Company. The gas stations and local business owners donated fuel and money to the Hongsa fire service to support their activities. In the education sector, DRR simulation exercises were conducted in some schools. The Child Club was established for a number of purposes including DRR promotion. Members of the Child Club include selected primary and secondary school students. Once these students have been trained on DRR activities, they conduct DRR activities at the community such as the creation of posters, risk maps, and evacuation plans. With these activities, Xayaboury Province received an award as the Champion of the School Safety in Southeast Asia given by the Association of Southeast Asian Nations (ASEAN) Socio-Culture Community in February 2017.

### Theme 3. Impact of policy implementation

Majority of school level respondents opined that students became more aware about DRR than before. They were able to explain the importance of DRR to their family members and community. For example, a DRR teacher said, “During the windstorm, I noticed that our students stayed in the school even after classes and only went home after it has passed.” The DRR curriculum was developed and DRR drills were conducted. In some schools, a DRR risk map was created, and emergency evacuation areas were defined with directions on how to reach these areas. In the community, the DRR emergency response unit was established and volunteers participated in it. Moreover, the basic warning system was set up (village speakers and whistles). Also, community evacuation areas were defined.

### School profiles

Out of the eight schools studied, six were project schools (Table 3). The MOES and SCI implemented a project in these schools. In these project schools, DRR lessons had more details for each disaster. The corresponding DRR teachers attended capacity-building trainings and were oriented on how to use the DRR textbooks.

**Table 3:** Characteristics of study schools related to disaster risk reduction

	School ID							
	1	2	3	4	5	6	7	8
The DRR lessons/curriculum that was designed by the project is implemented.	✓		✓	✓		✓	✓	✓
A DRR teacher who was trained by the project exists	✓		✓	✓		✓	✓	✓
Pilot school of the project	✓		✓			✓	✓	✓
Experience of fire	✓		✓	✓		✓		✓
Drills and exercises			✓	✓				
Assembly point	✓	✓	✓	✓	✓	✓	✓	✓
Fire equipment	✓			✓				
Emergency direction sign			✓					
Water supply	✓		✓					
Automatic circuit breaker			✓					
Fire inspection								

Among the eight schools, five have experienced fire disasters. All study schools have DRR classes which are offered approximately 4 h per month. Two schools have fire-fighting equipment. Two schools have conducted DRR simulation exercises. Also, all of the schools have an assembly point and are easily accessible by a vehicle. However, all study schools have not undergone fire safety inspections.

### Student knowledge on fire disaster

In every study school, only a small proportion of students (ranged from 7.2% to 35.0%) were able to correctly respond to all items under category of fire prevention. However, there was one item that almost half of students were able to correctly respond (51.2%) in this category. When the level of student knowledge between project and non-project schools were compared by Fisher’s exact test, students in the project schools were significantly more likely to know “causes of fire” (40.9% vs. 15.4%), “preventive measures against fire” (54.6% vs. 39.8%), “emergency telephone numbers of fire service/police station” (11.4% vs. 1.0%), and “how to use a fire extinguisher” (25.7% vs. 4.5%). Whereas, they were significantly less likely to know the fact that fire can happen anytime anywhere (16.6% vs. 39.3%) (Table 4).

**Table 4:** Student knowledge on fire prevention and response between project and non-project schools

Student-based survey item	Total (*n* = 869)	Project vs. non-project schools				*p value* ^a^
		School ID: 1, 3, 4, 6, 7, and 8		School ID: 2 and 5		
		*n* (*n* = 668)	%	*n* (*n* = 201)	%	
I. Fire prevention						
Do you know that fire can happen anytime anywhere?						
Yes	22.0	112	16.8	79	39.3	
No	78.0	556	83.2	122	60.7	< 0.001
Do you know what can be the cause of fire?						
Yes	35.0	273	40.9	31	15.4	
No	65.0	395	59.1	170	84.6	< 0.001
Do you know what to do when the fire occurs?						
Yes	51.2	365	54.6	80	39.8	
No	48.8	303	45.4	121	60.2	< 0.001
Do you know the emergency telephone number of fire service or police station?						
Yes	7.2	76	11.4	2	1.0	
No	92.8	593	88.6	199	99.0	< 0.001
Do you know how to use fire extinguishers?						
Yes	20.8	172	25.7	9	4.5	
No	79.2	496	74.3	192	95.5	< 0.001
II. Fire response						
What should you do when fire occur in your school?						
Report to the teachers or school principal						
Yes	97.1	657	98.4	187	93.0	
No	2.9	11	1.6	14	7.0	< 0.001
Call to fire service (police)						
Yes	92.1	614	91.9	186	92.5	
No	7.9	54	8.1	15	7.5	0.882
Switch off all electrical equipment						
Yes	93.2	620	92.8	190	94.5	
No	6.8	48	7.2	11	5.5	0.522
Evacuate the place						
Yes	84.7	577	86.4	159	79.1	
No	15.3	91	13.6	42	20.9	0.014
Fight the fire						
Yes	8.7	68	10.2	8	4.0	
No	91.3	600	89.8	193	96.0	0.006
Raise an alarm (shout out loud)						
Yes	90.9	605	90.6	185	92.0	
No	9.1	68	9.4	16	8.0	0.578
Gather in assemble point						
Yes	85.0	583	87.3	156	77.6	
No	15.0	85	12.7	45	22.4	< 0.001

^a^Fisher’s exact test

More than 80% of students were able to correctly choose appropriate responses in the category of fire response (ranging from 85.0% to 97.1%). Comparing between the project and non-project schools, students in the project schools were significantly more likely to know an appropriate response on “reporting fire to teacher” (98.4% vs. 93.0%), “evacuating during a fire” (86.4% vs. 79.1%), and “gathering at the assembly point” (87.3% vs. 77.6%). Whereas, they were significantly less likely to know an appropriate response on “fighting fire” (89.8% vs. 96.0%).

## Discussion

The National Strategic Plan for Disaster Risk Management 2003–2020 and the National Disaster Management Plan 2012–2015 were disseminated and implemented in most areas of the study provinces. Most facilitating factors and barriers were common in each level across sectors. Based on the three themes, effective coordination and ownership among the national DMC members, and strong support from the central government were perceived to be key facilitating factors for the policy implementation in the study areas. In contrast, unclear provisions in the national legislation, unclear mandates especially on leading the program, poor monitoring system, insufficient human resources, and lack of public-private partnerships were considered as barriers to the implementation of the DRR policy.

The lack of a national legislation on DRR appeared to be one of the major barriers identified. This problem led to unclear mandates resulting to weakness in stakeholder ownership and limited dissemination of the plans in some areas. This problem is similar to the experience of Nigeria during the development of their national policy on the Better Life and Family Support Programme [31]. The Xayaboury Province demonstrated the benefits of a having a policy in place. Its provincial government improved stakeholder ownership by coming up with clearly delineated roles and duties of each sector, creating an environment that enables policy implementation and making the program attractive to implementers. Previous studies also showed that the presence of legislations and practical guidelines are key factors for successful policy implementation of health promotion in Vietnam and Singapore [32, 33]. Currently, Lao PDR is drafting the national legislation for DRR.

One identified barrier is the frequent changing of lead agencies. It has caused confusion in terms of coordination and communication among implementers. It has also resulted to problems in supervision and feedback. Previous studies showed that implementation fails when policy development tasks are not well-defined [34, 35]. Similarly, schools failed to conduct DRR simulation exercises because it was unclear to which agency they should consult with. This has happened in similar situations in Singapore, England, and Australia [36–38].

There was also poor coordination between the provincial and district DMC. Coordination was only limited to responding to severe disasters. Also, there was no coordination during the other stages of the disaster cycle (prevention, preparedness, and rehabilitation). This can lead to some task redundancies, operation delays, and missed opportunities to conduct DRR activities. Apart from Xayaboury Province, there was a lack of public-private partnership across the study areas. These collaborations may introduce technology and innovation through improved operational efficiency; improve human resources such as capability building through training or academic exchange; and provide financial support. Successful public-private partnerships on health promotion have been observed in Cambodia, Vietnam, and the Philippines [39].

Monitoring was also lacking since the institution of the policy frameworks. Only Hongsa District, Xayaboury Province monitored their progress which facilitated success. Monitoring analyzes intermediary results and leads to mid-course revisions [40]. This also provides evidence of progress that is a significant source of motivation among implementers to sustain their practice [41]. The establishment of an internal or external monitoring and impact assessment system is necessary to ensure dynamic and strategic implementation of evidence-based policies [40–42].

The lack of competent human resource was also identified as a main barrier. This is usually because of the reassignments of trained personnel to other positions or even other institutions. This is similar to the experiences of both Lao PDR and Thailand in implementing school health policies [43, 44]. It can be addressed by training school health teachers on DRR. These trainings have been demonstrated to be effective in China [45]. Also, using the school cluster approach can aid in capacity building [46, 47]. School clusters are groups composed of several schools within a same geographical location for economic, pedagogic, administrative, and political purposes [48].

Xayaboury Province demonstrated strong leadership in the implementation of the policies. This has led to the development of local legislation, effective management of resources, mid-level coordination, and program monitoring. Moreover, it was the only province that developed public-private partnerships primarily for capability-building. Xayaboury Province was one of the pilot sites of the DRR project through the partnership between the government of Lao PDR and Save the Children International. This might have motivated the provincial leaders to have and maintain strong leadership. Also, the province established disaster legislation which identified clear and delineated tasks for each sector. Each sector was assigned district DRR areas that they will be responsible of. This promoted leadership and coordination at district levels. It will be beneficial if the experiences and lessons learned by Xayaboury Province be shared to other areas across the country. Sharing can be done through academic exchanges such as DMC lessons learned meetings and providing opportunities for DRR implementers from other provinces to observe how Xayaboury DMC practices may beneficial to them in terms of improving their knowledge and skills on DRR management. These have been proven to be successful in implementing the health promoting schools approach in Canada [49], and improved capacity-building and skills development among overseas professionals of Afghanistan, China, and the Philippines [50].

Students’ knowledge on items on fire response was higher, compared to those about fire prevention. The results of the current study can be explained by the content of DRR textbooks and the capacity-building activities for teachers. The books mainly centered on disaster response. Furthermore, teacher trainings also focused on this topic. These materials were developed in 2014. Hence, there is a need to review and eventually revise the current textbooks and teacher training programs.

Although the knowledge levels are still low on both groups, students among project schools had significantly higher knowledge level on fire prevention. Note that intervention activities include textbook revisions, teacher trainings, and simulation exercises. This can be seen as complementary to the current textbooks which focus only on fire response. It also highlights the importance of partnerships with NGOs. Therefore, continued collaborations with such institutions will be beneficial not only among schools but also with government agencies as well.

The study revealed that only 11.4% of the students know the emergency phone number for fire service even though these were mentioned in the textbooks. Some schools even posted these in their notification boards. This percentage is lower compared to the results of a study in Tanzania which showed that 18.0% of students know the emergency phone number [22]. The result of the present study highlights the need to improve disseminating the emergency phone numbers through posting these in each classroom and other areas of the buildings.

Interestingly, the student knowledge in the non-project schools was better in some items. One possible explanation is the experience of the DRR teachers. Some teachers came from schools who have experienced fires. Their experiences might have influenced how they teach DRR lessons. Previous studies also showed how teachers’ experiences impact students [51, 52]. Sharing of experiences among teachers can also be a good strategy to improve their knowledge and eventually communicate this to their students. One possible avenue is during weekly student gatherings.

There were two limitations that could have influenced the results of the study. Firstly, some of the key informants were newly designated, which might have provided a limited description of the situation of their organizations or schools. Secondly, the study schools were pre-selected by the district DMC which could have introduced bias. Because the present study selected schools with fire experience, this might have contributed to overestimating the level of school preparedness against fire and student knowledge. For example, a study conducted in USA demonstrated that those who have experienced a disaster more likely had better for disaster preparation [53].

## Conclusion

This study showed that although the policy did not reach some rural areas, it was widely disseminated and implemented in the study sites. The implementers had difficulty in implementing the policy because of unclear provisions in the national legislation, unclear mandates, poor monitoring system, insufficient human resources, and lack of public-private partnerships. However, these barriers can be overcome through strong leadership, ownership, and coordination, as demonstrated by one study province. Additionally, strong support from the central government can help promote policy implementation. All the study schools conducted DRR activities such as providing DRR classes and designating an assembly point, while some schools additionally conducted evacuation drills, trainings, and installation of fire equipment. Most of the students knew how to appropriately respond to fire. These results confirmed that the policy was actually implemented at school and reached students. However, the observed gap in DRR activities and student knowledge between project and non-project schools suggests that there is a need to improve the current school-based DRR program. The establishment of a legislation can lead to better implementation of the policy in Lao PDR.

## Abbreviations

ASEAN: Association of Southeast Asian Nations (ASEAN)
DMC: Disaster Management Committee
DRR: Disaster Risk Reduction
Lao PDR: Lao People’s Democratic Republic
MOES: Ministry of Education and Sports
NDMC: National Disaster Management Committee
SCI: Save the Children International
UNDP: United Nations Development Program
USAID: United States Agency for International Development
